# Equity and empowerment effects: Multiple styles of ‘voluntarism’ in community-based health projects

**DOI:** 10.1016/j.worlddev.2023.106448

**Published:** 2024-02

**Authors:** Carly Nichols

**Affiliations:** Department of Geographical and Sustainability Sciences, University of Iowa, 312 Jessup Hall, Iowa City, IA 52242, USA

**Keywords:** Volunteering, Gender, Nutrition, Ethics, South Asia, Self-help groups

## Abstract

Community health workers (CHW) are individuals with no formal health training who perform various roles to address health disparities. There are long-sustained debates over how different forms of incentives shape CHW programs, which are often staffed with volunteer or minimally remunerated women. These debates are complicated by the diversity of CHW roles and contexts in which they work. Evidence is particularly scant around “change-agent” style CHWs, who shape health knowledge and norms within their community. This paper addresses this gap through an analysis of a change agent-staffed program that provided nutrition participatory education through women’s groups in three eastern Indian sites. We examine how contextual factors across sites shaped change-agent management, and analyze the implications of each approach for efficacy, empowerment, and equity. Analyzing 68 interviews and 10 focus groups this study advances a typology of ‘varieties’ of voluntarism that we name laissez faire, active-cultivation, and honorarium-accountability, and uses comparative analysis to examine the equity and empowerment effects within selection, management, and payment. First, we find tensions in the community-based selection of volunteers because rather than selecting highly motivated women, groups selected women in the most favorable socioeconomic position to volunteer. Second, there is a tension around responsibility and expectations in that greater training and responsibility leads women to see more psychosocial empowerment (e.g., knowledge, confidence), but also may create more ‘costs’ to participation and leads to wider economic inequities in change-agent ranks. Third, we observe a misplaced focus on payments as central to change-agent motivation. While the two volunteer-only sites see payment as ‘the answer’ to motivation problems, the honorarium site sees payments as ‘the problem’ because they attract less intrinsically motivated individuals. We conclude that while payments may not make an unmotivated volunteer into a motivated one, this analysis suggests payments would potentially allow more marginalized women to participate, which may be key to making more equitable and efficacious impacts.

## Highlights

• I study a women’s group-based health social and behavior change intervention implemented by CHWs in three unique sites.
• I create a typology of volunteer CHW management ‘styles’ and use them to understand CHW empowerment and equity in each site.
• Giving volunteers more responsibility leads to greater empowerment, yet demands more time, and excludes marginalized women from being CHWs.
• Though payments are seen as ‘solution’ in volunteer sites and ‘problem’ in paying ones, I find they have little impact on CHW efficacy or motivation.
• However, I find payments may allow more marginalized women to be CHWs and thereby make the program more effective and equitable.

## 1 Introduction

Community health workers (CHWs) – are individuals – often women – that receive some health-oriented training yet have no formal health accreditations. CHWs are recognized as essential to ending nutrition and health disparities and can take many roles from serving as peer educator to performing basic preventative and curative health tasks to acting as a “change agent” to mobilize communities around changing health norms and addressing social determinants of health (WHO, 2018). Although there is a rich literature on factors that influence CHW efficacy, there is relatively sparse research examining how CHWs who function as “change agents” might be successfully deployed (Scott et al., 2018: 13). Over the last decade, however, governmental and non-governmental organizations (NGOs) have increasingly begun to use India’s large networks of women’s groups as platforms for participatory interventions to address everything from nutrition (Kumar et al., 2018) to mental health (Joag et al., 2020) to maternal and child health (Prost et al., 2013), among others (Desai et al., 2020). These projects typically train local women to work as a type of change agent to facilitate participatory learning within groups, adopt new health behaviors to change social norms, and demand accountability from government entitlement programs (Desai et al., 2020, Kumar et al., 2018, Sethi et al., 2019). Although emerging evidence suggests that with quality training and supervision, group-based change agents have the potential to be highly effective due to their community embeddedness and intimate knowledge of socio-cultural contexts (Desai et al., 2020, Gram et al., 2020, Rath et al., 2010), given the wide-range of different types of CHW programs, there remain questions among group-based interventions and CHW programs more broadly over the role that financial and non-financial incentives should play in maximizing worker motivation in ways that are empowering, equitable, and ethical (Gugerty et al., 2018, Glenton et al., 2013, Kok et al., 2015b, Maes et al., 2015, Ormel et al., 2019). This paper addresses these questions through developing a novel typology of CHW voluntarism “styles” as an analytical frame to unpack the implications of incentive design on CHW empowerment and overall programmatic equity.

How incentives shape community workers’ motivation and empowerment has been a hotly debated topic in research from the global South (Jenkins, 2007, Maes, 2012, Kok et al., 2015b, Ormel et al., 2019). Researchers argue worker motivation is sustained through a mix of financial and non-financial ‘extrinsic’ incentives (e.g., payment, preferential treatment, in-kind transfers, new skills, social recognition) and ‘intrinsic’ incentives (e.g., bringing positive change, fulfilling religious obligations, engaging in personal growth) (Ormel et al., 2019). Some argue that providing payments may ‘crowd out’ intrinsic motivation and produce CHWs that are only ‘doing it for the money,’ which may subsequently lead to a negative community response (Glenton et al., 2013, Ormel et al., 2019: 8). This work argues that voluntarism is viable because investing in CHWs’ capacity with quality training and supervision can lead to increases in knowledge, self-efficacy, and social prestige, which women may find more empowering and personally ‘valuable’ than small financial incentives (Glenton et al., 2013, Kane et al., 2016, Kasteng et al., 2016). Such arguments are attractive, particularly if there are fears that providing payments will lead to lack of sustainability (B-Lajoie et al., 2014) or if there are culturally-rooted commitments to voluntary service (Jakimow, 2010). Nonetheless, other evidence suggests lack of appropriate pay is the major cause of CHW attrition and demotivation (Kok et al., 2015b, Pallas et al., 2013) and that volunteers’ poor accountability makes it difficult to meet timebound goals (Ormel et al., 2019). Others offer a broader critique, arguing that promoting voluntary work through emphasizing the pro-social motivations risks reinforcing cultural narratives that position women as selfless, altruistic, and agreeable, and thus may perpetuate gender inequities (Closser et al., 2019, Maes et al., 2015, Steege et al., 2017).

These debates are complicated by consensus that CHW programs are highly variable and context-specific, where incentives and management expectations should reflect local realities such as socioeconomic demographics, physical geography, and institutional histories, among others (Kok et al., 2015a). As such, while the World Health Organization’s (WHO, 2018: 47) recently revised CHW guidelines “strongly recommend” all CHWs receive financial incentives to “enhance motivation and avoid perpetuating gender inequities,” the authors quickly qualify that this recommendation “may not apply” to “dedicated volunteers who willingly perform their roles on a pro-bono basis.” They further concede the difficulty in drawing “a clear line” between willing volunteers and those volunteering for lack of livelihood options, so they conclude that local authorities should make the final decision based on local norms and CHW effort. While commendable for acknowledging that ‘context matters’, these guidelines leave unanswered questions around what contextual factors might support volunteer or remunerated work, particularly within group-based interventions where CHWs may already be a group member and only work a few hours per month. More pressingly, while the guidelines are concerned with gender equity and project efficacy, they offer few insights on how financial incentives (or lack thereof) may shape a program’s impacts on conceptions of *equity* and *empowerment* that go beyond simple considerations of gender. Yet examining how incentive structures shape equity (*who* can participate) and empowerment (does participation enable women to address structural marginalization) is critically important within group-based health promotion as well as CHW programs more broadly because these initiatives have been promoted precisely to address structural inequities and are often underpinned by commitments to collectivization to work towards economic, political, and social justice.

This paper addresses these gaps through using a framework that integrates conceptualizations of psychological and political forms of worker/volunteer empowerment (Kane et al., 2016, Closser et al., 2019), with considerations of equity– examining *who* is able to participate in volunteer programs and how that might matter for project impacts (c.f. McCollum et al., 2016). This paper addresses two central questions:

1. What contextual factors shape the potentials and limits of change agent style volunteer programs?
2. Are there tradeoffs in terms of efficacy, equity, and empowerment within different styles of volunteer change agent management?

I present an analysis of qualitative data collected from participants in a volunteer-led nutrition program implemented through women’s groups in three blocks of eastern India. The data are unique because the implementing NGO, PRADAN, allowed each block team substantial autonomy to manage volunteers in ways that reflected contextual factors such as socio-demographics, human development, and local histories.

Our analysis contributes to several debates in the literature. First, building off earlier work (Nichols 2021a), we find site-specific differences in institutional social capital and socio-economic development had dramatic impacts on each block’s approach to change-agent management. Second, acknowledging voluntarism as a fluid, socially-defined concept that takes different forms in practice (Bailie-Smith, 2019, Jakimow, 2010), we advance a typology of three volunteer models to group-based health promotion: ***‘laisse faire’ voluntarism*** that adheres to ethical principles of working per one’s desire with low expectations for timebound action, ***‘active-cultivation’ voluntarism*** where significant effort is made to motivate volunteers and there are greater expectations for action, and ***‘honorarium-based voluntarism’*** where a smaller set of women are given small payments per training and face greater expectations. Using this typology, we find that each model manages ‘change agent’ responsibility, reward, and harm in different ways, with implications for efficacy, equity, and empowerment. We conclude by reflecting on three lessons policy makers and researchers can take from this analysis and ultimately argue that while payments may not make unmotivated volunteers motivated ones, they allow for more equitable and empowering health outreach.

The remainder of this paper proceeds in five parts. Section two outlines our conceptual framework and section three provides a description of the PRADAN intervention. Section four outlines the study sites and methodology. Section five consists of the analysis, presented in three vignettes of each voluntarism model. Section six discusses the results comparatively and concludes.

## 2 Lay health worker empowerment and equity

While much work has examined motivations, challenges, and experiences of CHWs to improve their efficacy, reduce attrition, and ensure sustainability, a slimmer body of research has focused on whether CHW programs are empowering women and whether their effects are equitable (Scott et al., 2018).

In looking at CHW empowerment, there have been two dominant approaches. Kane et al. (2016: 32) employ a construct from managerial theory (Lee and Koh, 2001) to examine CHW psychological empowerment through four lenses: competence, meaningfulness, impact, and autonomy. The authors probe CHW perceptions on whether they feel *competent* in their job, if they believe it to be *meaningful*, with a positive *impact* on the community, and if they have *self-determination or choice* in how they wish to perform the work. The framework approximates measures of program efficacy by looking at CHW’s perception of their competence – or ability to perform a task with skill – as well as whether they think their work has positive impact on the community. It closely aligns with motivational theory concepts on how the right mix of intrinsic incentives (e.g., meaningfulness, skill) and extrinsic incentives (e.g., social prestige, autonomy) are central to maximizing CHW job satisfaction and retention (Ormel et al., 2019). Yet, while Kane et al. (2016) find the framework useful to analyze CHW experience, they find their analysis falls short in understanding how context or design factors (e.g., incentives) shape the outcomes, except noting it is disempowering when workers feel ‘undervalued’. The authors further concede the framework provides a narrow view of empowerment that may serve more as managerial tool to assess worker motivation without conceptual space to question the relations of power in which health workers or volunteers are embedded.

Here it is useful to turn to Closser et al. (2019), who build on this assessment of psychological empowerment by arguing that social and political empowerment are equally important. They contend since CHWs are disproportionately resource-poor women who face socio-political marginalization it is critical that CHWs are also enabled to analyze the relations of power they are embedded in and collectively work to change them. (Closser et al., 2019: 300, c.f. Kabeer, 1999). This draws on Maes’ (2011) work that complicates simple notions of ‘choice/self-determination’ and ‘meaningfulness’ through demonstrating how gendered and religious tropes of moral duty are invoked to encourage women’s participation and impress upon women the ‘naturalness’ of them fulfilling these duties. Closser et al. (2019) finds similar dynamics and argues that in Ethiopian CHW programs women’s agency is not expanded but rather constrained in that she has little latitude to flout gender norms that emphasize altruism. Whereas women may not be *forced* to work, they may feel that they will face social penalties if they do not oblige (see also Nichols 2021a). Closser et al. (2019) call for a broader conception of empowerment within CHW programs where women are engaged in processes to empower them *to be effective workers and also* processes that allows them to question and shift the socio-political relations of power in which they are embedded.

In practice, this means CHWs have the ability to challenge organizational policies that they feel limit their ability to volunteer/work in ways that respect autonomy and dignity. Methodologically, broadening Kane et al.’s (2016) framework to include social and political empowerment entails a closer look at *process* in understanding the ways that CHW perceptions of meaningfulness and self-determination are forged as well as, I argue, foregrounding questions of ethics and equity. Taking an ethical lens means asking how visions of voluntarism are operationalized and whether improved health outcomes (“the ends) justify using poor women’s unpaid labor (“the means), especially when organizations “actively shape volunteers’ motivations” (Maes, 2012). An ethical approach recognizes that voluntarism takes diverse socially-defined shapes across different cultural contexts yet demands rigor in understanding the particularities within specific cases (c.f. Baillie Smith et al., 2020). For example, Jakimow (2010) describes how sociocultural notions of *sewa* – Hindi for service –a respected South Asian politico-religious practice and feature of the Gandhian independence movement – are now conflated with Euro-American notions of voluntarism that mean ‘working for free’ or ‘according to one’s volition’. Jakimow notes that historically, *sewa* consisted of individuals freely doing service with knowledge their basic needs would be met through community. Her analysis reveals that while contemporary development agencies invoke *sewa* to gain legitimacy among the communities in which they work, the ways voluntarism is operationalized within timebound projects often necessitates a deviation from its roots of organic acts of charity. Using an ethical lens, thus, helps to examine the processes used to recruit individuals and whether principles of volunteer self-determination are upheld in allowing volunteers to work to the degree they wish.

Volunteering may be ethically sound without addressing the needs of the most socio-economically marginalized. While much CHW literature focuses on distributive equity within beneficiary population (e.g., the neediest receiving preferential resources) (Houweling et al., 2013; George et al., 2018), there has been less analysis of whether opportunities to be CHWs are also equitably shared (McCollum et al., 2016). This is critical as McCollum et al. (2016) find that when programs recruit women who are socioeconomically marginalized, they tend to be more inclusive of other marginalized households. Moreover, while the goal of CHW programs is to improve population health equity, research suggests that oftentimes CHWs receive the greatest rewards (through intrinsic/extrinsic benefits) in “information-centered” interventions even when the impact on population health is null (Chandra-Mouli et al., 2015, Gilbertson, 2020). Thus, it is critical to examine how opportunities are distributed, and whether programs emphasize *recognitional* equity in their opportunities by acknowledging how different *contexts* such as education, ability, or economic status may shape women’s ability to participate and providing special accommodations for CHWs who may need more support (c.f. Fraser, 2009).

Our conceptual framework thus aims to illustrate how the three components of efficacy, equity, and empowerment interact under three different management contexts of the same program. It presents an interesting set of case studies to compare how an identical curriculum was carried out through voluntary/minimally paid workers in different manners that align with the socio-ecological context of the sites.

## 3 Description of PRADAN intervention

PRADAN has worked for over 30 years in socioeconomically marginal areas of eastern and central India to mobilize networks of women’s saving and credit groups (known as self-help groups or SHGs). In 2013 PRADAN partnered with the Public Health Resource Society (PHRS) to develop and implement a program that addresses health and nutrition in three pilot sites, which expanded to eight in 2015. The program, ‘Facilitating Action Against Malnutrition’ (FAAM), used rigorous community needs assessment to iteratively design a set of nine participatory learning modules that each featured a story about a woman character, who faced a particular health challenge (e.g., low dietary diversity, anemia), which she eventually overcame through collectively addressing social determinants of health with practical actions (e.g., cultivating nutrition gardens, vowing to eat complete meals with protein and vegetable, negotiating work parity with her husband). (see Nichols 2021b for full description).

In fall 2016, PRADAN block sites assembled teams of paid and volunteer CHWs to facilitate modules in SHGs. Each block was led by three college-educated PRADAN/PHRS staff members trained in rural development or public health and a team of 4–6 locally recruited nutrition “mentors” trained to work as field-level supervisors. After attending a three-day orientation, mentors and PRADAN executives held village-level meetings of SHG members to facilitate the selection of one woman to serve as a “change vector” (CV). CVs were expected to attend a three-day residential training and subsequently attend centralized refresher meetings each month thereafter. With mentor support, CVs were expected to tell stories and facilitate discussions from FAAM learning modules among the SHGs in their hamlets (approximately 2–7 SHGs/hamlet), while also working as ‘change agents’ by adopting optimal health and nutrition practices, speaking informally about health in everyday encounters, and mobilizing women to enact these changes in their own communities.

PRADAN’s vision of voluntarism was informed by their observations that when they paid SHG women to facilitate trainings, then activities stopped when the project (and payments) ended. Moreover, they had witnessed SHG members demonstrate voluntarism in helping other women form SHGs or organizing around issues such as stopping alcohol sales (Pers. Communication). Thus, PRADAN felt that making the CV a volunteer position would be optimal, particularly since they would only need to work a few hours per month in their immediate hamlet. Because there were no monetary incentives, program designers assumed that women would self-select based on their *intrinsic* motivation (e.g., desire for knowledge or to bring change) or to receive *extrinsic* incentives (e.g., social prestige) rather than income or desire for career advancement (c.f. Glenton et al., 2010). Thus, PRADAN hypothesized that if they could identify a woman in every hamlet who was strongly motivated to be a ‘change vector’ for nutrition, then community mobilization would continue beyond project end.

PRADAN is organized as a federation, where block-level teams comprised of six college-educated professionals have relative autonomy to design community engagement to fit site-specific contexts. Thus, although FAAM was a centrally designed intervention, there was significant room for local innovation at the block level to adapt to community needs.

## 4 Methodology

The study was conducted in March-June 2019 as a nested qualitative evaluation within an International Food Policy Research Institute (IFPRI) quantitative impact evaluation of FAAM. While IFPRI midline data collected in 2017 found CV knowledge of curriculum was high and many modules were transacted, SHG members ‘retention’ of messages remained relatively low. This study was designed to use qualitative methods to understand more nuanced processes at the levels of the teams, CVs, and groups as to explain these findings.

### 4.1 Site selection

The three study sites of Purulia (West Bengal), Bastar (Chhattisgarh) and Pashchim Singhbhum (PS) (Jharkhand) were selected based on site attributes, project evaluation midline data, and consultation with PRADAN and PHRS.

Bastar and Purulia were selected as primary data collection sites since both implemented FAAM similarly, recruiting local salaried mentors in 2016 to support a cohort of approximately 75 volunteer CVs that were selected by SHG members. Midline data alongside PRADAN’s internal reporting suggested that Purulia was a ‘high-performing’ site, while Bastar was lower performing based on number of modules transacted in intervention villages and community members’ retention of central messages. For example, one of FAAM’s messages related to dietary diversity was that individuals should eat ‘tri-color food’ (carbohydrate, protein, vegetable) yet only 35% of Bastar respondents reported hearing this in their SHGs, compared to 61% in Purulia.^1^ Block reporting suggested FAAM was more active in Purulia, and PRADAN staff felt Purulia was a ‘strong’ site since it is one of PRADAN’s oldest sites, had relatively mature women’s institutions and had been successful in livelihood projects such as cash-cropping vegetables. Conversely, Bastar district was newer with younger women’s institutions, yet spent significant resources expanding over the last three years under the government’s National Rural Livelihood Mission scheme. PRADAN’s agricultural development focus was less successful in Bastar, which staff credited to the Scheduled Tribe (ST, or *Adivasi* in Hindi) population that was less engaged in agriculture, relying more heavily on forest-based livelihoods and daily labor.

PS was selected for limited data collection because from 2015 onward it served as a pilot site, where PRADAN experimented with different program designs that did not include the position of a mentor to support CVs. Responding to CV requests, the PRADAN team provided honorariums of Rs 125 (US $2) to a group of approximately 30 CVs for each training conducted with 15 + women. While staff instituted mentors in 2016, they continued their practice of remunerating CVs. Despite using paid staff, PS also had relatively low exposure to key messages (e.g., 38% of midline respondents heard about ‘tri-color food’), thus it was selected to more specifically examine how the use of payments may have impacted CV motivation and experience. PS was also unique because it was the site of ongoing Naxal conflicts, making safety and mobility preeminent concerns. Women’s institutions had been hampered by Naxal tension, and while they were older than in Bastar, they had not reached the maturity of Purulia and remained dependent on PRADAN for support.

While these areas diverged considerably in PRADAN’s institutional history, there were also major demographic and cultural differences across sites. PS and Bastar were comprised almost entirely of *Adivasi* populations that speak their own distinct tribal language and have low levels of literacy (Table 1). While PRADAN members had basic proficiency in understanding Tribal languages, they were not conversant in them, and had to use bi- or tri-lingual SHG women as interpreters. Conversely, Purulia is dominated by lower caste Hindus proficient in Bengali and with higher levels of literacy. In terms of basic geography, PS and Bastar are characterized by forested, hilly tracts with low population densities, remotely situated villages and poor road access whereas Purulia was flatter with better road connectivity and higher population densities. Government coverage of basic health interventions also varied considerably across sites (Table 2).

**Table 1:** Block Level Census Statistics and Characteristics (Source: Census of India (2011).

District (State)	Block 1, Purulia (West Bengal)		Block 2, Bastar (Chhattisgarh)		Block 3, Pashchim Singhbhum (Jharkhand)	
	*Total*	*Female*	*Total*	*Female*	*Total*	*Female*
Population	137,143	67,048	79,360	40,389	77,697	39,085
Children (Age 0–6)	20,832	10,116	13,445	6,634	13,287	6,616
Literacy Rate	66.2 %	43.8 %	38.3 %	24.8 %	59.2 %	36.4 %
Scheduled Caste^1^	12.4 %	12.3 %	0.3 %	0.3 %	5.8 %	6.0 %
Scheduled Tribe^2^	11.4 %	11.4 %	82.9 %	83.2 %	61.2 %	61.7 %

Data from Census 2011.

1 Scheduled caste is the name given to the most marginalized castes in India (also called Dalits or untouchables). According to Census of 2011 they comprise approximately 16.2% of the total Indian population.

2 Scheduled tribe is the name given to 650 + unique ethnic groups in India often viewed as indigenous peoples (also called *adivasis)* and who have faced systematic marginalization. According to the Census of 2011, the tribal population comprises 8.6 per cent of the country’s total population.

**Table 2:** District level healthcare coverage and basic infrastructure (Sources NFHS 4).

	Purulia, WB	Bastar, CH	Paschim Singhbhum, JHK
Coverage of key maternal and child nutrition services			
Health and nutrition education from *anganwadi* during pregnancy^a^ (%)	47.8	78.4	12.6
Health and nutrition education from *anganwadi* while breastfeeding^b^ (%)	39.2	78.5	12.0
Breastfeeding counselling (%)	68.5	70.0	12.8
Counseling on child growth (%)	50.4	66.2	5.3
Basic Infrastructure and geography			
Population density (persons/sq. km.)	408	135	208
Households with electricity (%)	78.1	83.8	64.1
Households with improved drinking water (%)	81	93.8	64
Households with improved sanitation (%)	8.2	11.9	10.3

^a^ Percentage of women (15–49 years) with children under 5 years of age who received health and nutrition education or advice on breastfeeding from the AWC, when they were pregnant or breastfeeding when they were breastfeeding their youngest child.

Data from NFHS 5.

### 4.2 Sampling and data collection

The study used the same purposeful sampling in the primary data collection sites, Bastar and Purulia, and a modified version in PS. In all sites, we conducted interviews with three core PRADAN/PHRS staff. In Bastar and Purulia we conducted both semi-structured interviews and focus group discussions (FGDs) with all mentors. In all sites, CVs were selected for interviews and FGDs in collaboration with PRADAN using data from internal CV grading sheets^2^ to select CVs that ranked both high and low and also to achieve even geographic spread across the block (e.g., selecting some closer to towns and some remotely located). In PS, due to limited time and mobility because of Naxal tensions we conducted an FGD with mentors and three FGDs with CVs.

The CVs all had basic primary education and literacy skills, except for in Bastar where two CVs were illiterate. Similarly, the majority of CVs were married with children, although in Bastar and PS there were both two unmarried younger CVs, respectively. CVs caste/ethnicity largely mirrored their areas where Purulia CVs were all OBC and Bastar and PS CVs were majority *Adivasi*, with each site also having two CVs identifying as OBC. While we did not ask for detailed data on household economic status, all households practiced diversified livelihoods that included semi-subsistence cultivation for consumption and local marketing, along with daily wage labor. As noted above, Purulia had a higher prevalence of economically advanced households as PRADAN has had success in promoting cash-cropping here.

We collected data through both semi-structured interviews and FGDs (Table 3). Interviews and FGD guides were written in English and translated by native speakers of the local language. We piloted and revised guides in Purulia prior to data collection, which took place in May – June 2019. The interviews consisted of asking respondents (either mentor, CV, or PRADAN/PHRS staff) to describe their selection, training, and job role as well as to tease out motivations for doing FAAM work. We asked respondents to tell us about parts of the curriculum to assess their knowledge and invited them to express challenges and bright spots of both their particular job and the FAAM intervention. The FGD discussions consisted of similar questions yet gave CVs or mentors opportunity to build off each other’s thoughts and agree or disagree (Hay 2000). The data research managers in Purulia and Bastar transcribed and translated interview and focus group audio, which were quality checked by third party native speakers. In total 64 interview and 6 focus groups transcriptions were uploaded to MAXQDA 2018 qualitative data software for analysis.

**Table 3:** Total number of Respondents, by Study Site.

Type of Respondent	Purulia	Bastar	Paschim Singhbhum	*Total*
PRADAN Block Professionals	2	3	3	*8*
Mentors	5	4	7	*16*
Change Vectors	23	19	11	*49*
SHG Members	14	13	0	*27*
*Total*	*44*	*39*	*21*	***100***

Within interviews, a limitation was social desirability bias, where respondents answer questions in ways they think will be socially appropriate (Dunn, 2010). This was particularly potent here, as there were sensitive questions about job performance, satisfaction and remuneration. To help minimize this bias we spent time building rapport with each woman and emphasized that their experiences were important so that projects like FAAM could be improved. At multiple times we reminded women interviews were anonymous and confidential to keep space open for candid reflection. My positionality as a ‘outsider,’ particularly a white, American woman, undoubtedly shaped the research interaction, and I used practices of reflexivity – i.e., nightly debriefings with the research team- to minimize power dynamics and check cultural assumptions. The limited data collection in PS is a weakness in our study, however, we are confident in the data as across all three CV focus groups we received similar responses, which triangulated with mentor and staff data. Finally, the cross-sectional data represents a limited ‘snapshot’ of an intervention designed to plant the seeds of longer-lasting mobilization around nutrition and health. There is a need for future research on community-engaged health mobilization to employ longitudinal studies to track the ways subjectivities and practices evolve after engagement in these groups (Gugerty et al., 2019).

To answer the research questions, I used a two-cycle structural coding approach, first parceling transcripts into relevant domains such as selection experience, motivation, or volunteer challenges. The second cycle of coding analyzed variability within each structural code using inductive, in-vivo codes that worked to capture themes in respondents’ own language (Saldaña, 2011). After developing the codebook, the coding schema was crosschecked by an IFPRI researcher trained in qualitative methods for accuracy and precision. I also frequently communicated with members of PRADAN to receive their feedback on preliminary analysis, however they were not formally involved in analyzing the data. We organized coded data into a CV profile matrix to examine relationships between different factors (e.g., motivation and challenges) within a single respondent, and to examine variability within factors across responses. From this analysis we quickly saw significant differences between women’s experiences with training and their reports of benefits and challenges or harms that accrued through work. We triangulated these findings with interview data from mentors and PRADAN staff to pinpoint specific elements of the program design and site context that shaped both potentials and limits to CV work in each block.

## 5 Findings

Our analysis found that each block team employed different management strategies to maximize CV effectiveness and minimize CV attrition through negotiating a balance of reward, responsibility, and ‘costs’ of CV engagement. While all teams strived to implement FAAM as designed, they iteratively adapted their management strategies in response to site-specific factors such as human development and PRADAN’s institutional history. While these strategies were logical responses to site-specific conditions, they also yielded clear trade-offs in terms of efficacy, equity, and CV empowerment that we describe below in three short case study vignettes.

### 5.1 Bastar and laissez faire voluntarism

In Bastar, PRADAN’s relatively short institutional history alongside linguistic and literacy challenges significantly shaped program potential. While staff initially recruited a large cohort of CVs through village level selection processes, they saw nearly every CV ‘drop out’ shortly after the first orientational training. A PRADAN leader explained that massive dropout occurred firstly because families did not support women leaving for unpaid work, but also because the 3-day initial training was insufficient in motivating CVs.

One of the biggest gaps we had in the design was…the 1–3 day long trainings may not have really, you know, inspired [the CVs] to a level where they will voluntarily reach out to other members. This would have required a lot more focused investment on them (PRADAN interview, 6/24/2019)

Notably, for most CVs, this was the first time they had served as trainers for PRADAN since SHG-related work was still nascent in many villages. Thus there was less social capital between PRADAN and the community that program staff could draw on, particularly since women had not yet seen economic benefits from SHG involvement (see Nichols 2021a, also Gope et al. 2019 for evidence that pre-formed SHGs may not be the best site for health promotion). In response to initial attrition, Bastar staff triaged the program by hand-selecting women they knew had the time and language skills to complete the work. Due to a scarcity of literate SHG members, several women were selected who were illiterate or unmarried youth not part of an SHG. While CV respondents said they were motivated to take the role to “bring change” or “improve their own knowledge,” nearly one-quarter could not express a clear reason for why they chose to be CV other than that they were asked (Table 4). This introduced a tension observed across all sites, where voluntarism did not necessarily mean working from one’s own will but rather consenting to one’s nomination by either a group of peers or a figure of authority.

**Table 4:** Motivations to accept role as change vector (CV).

Motivation	Purulia (n = 14)	Bastar (n = 12)	Paschim Singhbhum (n = 12)	Exemplary Quotes
Bringing change	5 (38 %)	5 (39 %)	5 (42 %)	I thought that situation of the village is not OK right now. If I do this work, then there might be some improvements in the village. Mothers were not taking care of their children properly. So, I thought that I would do the work (Paschim Singhbhum CV, 6/15/2019) Becoming CV, I can go to the Village Organization and be talking to the women members….… the members do not know much, so as a CV I could come to learn and tell them (Bastar CV, 5/25/2019b)
Knowledge (about oneself/health)	5 (38 %)	3 (25 %)	4 (33 %)	I thought that I will learn something. See, if I stay in my house, then I will become a frog in a well. But, if I go out, I can learn many things. That is why I wanted to become. (Purulia CV, 6/11/2019) I thought that if I join [as CV] then I can learn about all these and I will be able to better take care of my kid. I thought like this and I decided to join (Paschim Singhbhum CV, 6/14/2019b)
Meeting Others	2 (15 %)	0 (0 %)	0 (0 %)	When I go to SHG and talk to others, then I feel good. I thought that if I get to meet so many people, it would make me happy. That is why I thought of doing it (Purulia CV, 6/4/2019)
Previously worked	2 (15 %)	1 (8 %)	1 (8 %)	I thought that I was working as ‘*siksha-sathi*’ and so I already knew so many didis. So, I thought I could do it. (Purulia CV, 6/9/2019)
Sense of responsibility	3 (23 %)	0 (0 %)	3 (25 %)	I thought if everybody is fine and if I have to take responsibility, then I will work as CV (Purulia CV, 6/8/2019a) Yes. I also had agreed [to be CV]. I thought if everyone was agreeing, then I should do it. I thought that I would be able to devote time (Paschim Singhbhum CV, 6/14/2019)
No clear motivation	0	4 (33 %)	0	They told me to become CV, so I became….and I also felt desire to do. (Bastar CV, 5/27/2019)
Money/Good opportunity	0	0	4 (33 %)	I thought if I need something to buy, then I will be able to do it with the money (consensus from group) (Paschim Singhbhum CV, 6/15/2019)

### 5.2 Management

With a new cohort of CVs, staff tried to minimize future attrition by employing a flexible orientation to volunteer work, where they placed few expectations on CVs (e.g., CVs typically looked after only 2–4 SHGs), and mentors worked hard to respect CVs’ volunteer status. One mentor explained:

I also feel we are not giving them any money so we cannot force them to come and sit, so we work as per their pace and interest so for instance in one village if the meeting is not conducted we would go to another village (Mentor FGD, 5/31/2019)

Moreover, while Bastar initially held centralized ‘refresher trainings,’ staff soon observed that many women did not attend. CV respondents concurred they had attended few, if any, central trainings, opting to skip them to attend to other responsibilities or because they were wary to attend. For example, one CV explained,

They were telling about trainings, but I wasn’t going for them….There were CVs in other hamlets; they weren’t going either….As someone who didn’t know much about the training, why would I go alone?. Either would we have to go on our own expense or else they would come in vehicle to pick us up, and drop us back (CV interview, 5/30/2019b)

This report was striking as it illustrated the larger context of weak institutions and community ties, which shaped Bastar’s approach. While staff offered to provide transport to the training, the CV remained reluctant to attend since she observed others were not going. Yet, the Bastar team did not have time to address larger challenges of community mobilization within FAAM’s tight timeline. They, thus, modified their strategy, shifting from centralized meetings to individualized field-level refresher trainings that mentors gave to CVs directly prior to SHG meetings.

While we found this worked well to include illiterate women who needed special tutelage, or those who could not travel due to household duties, it also had drawbacks. The mentors explained these pre-meeting coaching sessions often resulted in the CV pleading with them to tell the story on their behalf. The mentors explained:

We go and say to the CV, “sister, please tell the story,” but she says, “no, I feel shy, you only come and tell the story I will tell any other time” (consensus from group, Mentor FGD, 5/31/2019)

This dynamic helped to illuminate our findings that Bastar CV respondents struggled remembering key parts of the curriculum (see Nichols, 2021b). Yet, though mentors felt the lack of collective training hampered CVs’ knowledge and skill-development, they felt the larger problem was that CVs had no opportunity to collectively express frustrations and successes to receive social support. The mentors explained that the centralized trainings held in early stages of FAAM, *“helped [CVs to] understand the difficulties they face in their work; and when they sat together, their problems come to the front”* (Mentor FGD 5/31/2019).

### 5.3 Benefits, costs, equity

These mentor’s insights were critical, as CV discouragement was one of the biggest challenges in Bastar. CVs often questioned their *competence* as well as the *impact* they had on the community, thus one Bastar professional lamented, *“in a lot of places, CVs have lost their motivation… Because in some places the SHG discussion is very less, so what will the CV do?”* (Bastar interview, 5/29/2019). While there was a broader context of poor SHG mobilization (see Nichols, 2021a), CVs often took the poor community response personally, which further eroded their confidence. In responding to a question about difficulties in CV work, one young CV says.

R: When SHG women do not understand we try to make them understand. [But] they sit in the circle and talk about random things and do not pay much attention

**I:** So they do not pay much attention.. why is it so?

**R**: It may be the way we explain the things is….(leaves sentence incomplete) because few understand and few does not so I feel it has to do with my way of explaining as wrong or right (CV interview, 6/24/2019b)

The ad-hoc system of field-level training and the poor community response meant Bastar CVs had more difficulty identifying non-monetary benefits of their positions than their counterparts in other sites (Table 5). Most CVs stated that “bringing change” was a positive aspect of their work, but few spoke about increased social status (only 8% compared to 71% in Purulia) or improved mobility/autonomy (only 8% compared to 36% in Purulia). However, notably, many CVs – particularly illiterate or young, unmarried women- spoke very fondly of field-level trainings and expressed pride in receiving individual tutelage on important issues. Thus, due to individualized investments in CVs, some, and perhaps the more marginalized, expressed appreciation for the program.

**Table 5:** Benefits Associated with CV Work Across Sites.^a^

Benefits	Purulia, n = 14^1^ (45 total responses)	Bastar, n = 12^2^ (21 total responses)	Paschim Singhbhum, n = 11 (4 FGDs) (26 total responses)	Exemplary Quotes
Prestige/Respect	10 (71 %)	1 (8 %)	3 (27 %) (2 FGDs discussed)	I get so much respect. When I go to someone’s house, they say “CV has come. Please have a sit here.” Other women will also come “CV has come”. Then I like it. (Purulia CV, 6/4/2019) Earlier [women] were not even agreeing to sit, but now that they see so much information coming, they do give us respect (Bastar CV FGD 6/1/2019)
Knowledge (about health & oneself)	7 (50 %)	4 (31 %)	5 (45 %) (3 FGDs discussed)	I have learnt many things. I was underweight during the initial BMI camp. Then I had changed my eating habit. Now, I have healthy weight. (Purulia CV, 6/7/2019) Since the time I have been involved with this, I am taking better care of my baby and the other women members of the house. I am taking more care of my food intake. (Paschim Singhbhum CV FGD 6/14/2019)
Bringing Change	7 (50 %)	8 (62 %)	2 (18 %) (I FGD and 1 interviewee discussed)	I felt good, because we have no information and therefore sometimes people lose their lives [during childbirth] too, Therefore it felt good to provide people the information. Because someone’s life could be saved. (Bastar CV, 6/3/2019) Earlier, mothers did not take care of their kids so much. Kids did not use to get proper vaccination. Women did not use to go to anganwadis. They did not get registered for schemes. Now we have come and tell these things in the village. It is good that we take care of our own village. (Paschim Singhbhum CV FGD 6/14/2019)
Mobility/Autonomy	5 (36 %)	1 (8 %)	5 (45 %) (3 FGDs discussed)	Yes, we are getting the chance to go outside. We are getting knowledge. We are learning. I like this. I can go to other blocks or bank which I like. I really like spending time with others. (Paschim Singhbhum CV FGD 6/14/2019) Now, I can go outside, I can go to bank and Panchayat. I can talk to officers. I was scared before. But, after all the trainings I can go and speak at the bank or speak with the officers. I am not scared anymore. (Purulia CV interview, 6/7/2019)
Meeting Others	4 (29 %)	4 (31 %)	9 (82 %) (3 FGDs discussed)	In one group there are more than 15 didis. So, I feel good when I talk to them. (Paschim Singhbhum CV FGD 6/14/2019b) We are getting to meet other women and we can talk to these women. This makes me happy. I can meet so many new people. Then I forget about my household work. (Purulia CV FGD 6/8/2019a)

a Responses to open question – ‘What are the best parts of being a CV?’ Respondents answered unprompted and categories emerged during analysis.

1 In one FGD all of the women mentioned and had consensus on all of these benefits except for gaining knowledge (6/8/2019b) whereas in the other FGD they concurred on all benefits except bringing change (6/8/2019a). In the frequency tabulation there is 1 point for each FGD—despite the fact that 5 women really agreed upon it.

2 One CV in Bastar identified more as the secretary and was unable to understand our question or provide a response. Like many CVs in Bastar, she was very minimally engaged in CV work.

Though Bastar CVs did not seem to derive much psychological ‘empowerment’ through their position, they notably never complained that the work interfered with their livelihoods or household duties.^3^ Perhaps because there were few expectations placed on them and mentors diligently respected their volunteer status, CVs had never raised the issue of payment to mentors, PRADAN or within interviews, or expressed resentment about voluntarism. This dynamic is notable, as it stands in stark contrast to the CV situations in Purulia and PS, where CV demands for payment were a perennial concern.

### 5.4 Purulia: Active cultivation and making change vectors ‘work’

Purulia’s long institutional history meant that block executives had good relationships with many communities and women had higher levels of human development. The PRADAN block leader stated over the years they had identified *“some accountable women who can do [training/facilitating work] so we have to rely on all of them and make them trainers for all the work”*. Yet though staff reported CVs were preferentially selected based on past experience, the CVs themselves largely reported they were selected because they had amenable home environments. For example, one woman said,

My husband will not create any problem to me. He is not a drunkard. I can go and spend time for trainings. Most of the women do not have time. So the other SHG members said ‘this woman does not have any problem in her house. Her husband is also good. So, she should do the work’ (CV interview, 6/11/2019)

Thus while, in theory, a goal of voluntarism is to attract women intrinsically motivated to do social work (c.f. Glenton et al., 2010), we observed that more logistical considerations are prioritized in practice. Moreover, similar to Bastar, we found that while 75% of selected women expressed intrinsic motivation for doing the work, the remaining 25% participated because they felt a responsibility to oblige with their selection.

However, while Bastar CVs were largely ‘working’ in PRADAN for the first time, many Purulia CVs had previously occupied other paid positions, thus there seemed to be a latent hope that the CV role would eventually become paid (see also Maes, 2011, Ormel et al., 2019). Thus, from project inception *“many CVs raised the payment issue”* and dropped out when PRADAN reaffirmed its commitment to voluntarism. One CV recounted the scenario in her village:

There was another CV, but she left midway. She told [staff] that she could not continue, as there was no salary. Then, another CV was selected. She continued for 3–4 meetings. Then, she also left. She said “it is better to do labor work. I have to get absent from work to go to Purulia and conduct these meetings, so my family is not permitting me.” (CV interview, 6/4/2019)

However, rather than adjust expectations as in Bastar, Purulia strategized to actively inculcate an ethic of voluntarism to continue an ambitious implementation agenda with CVs covering 5–10 SHGs. The team implemented monthly centralized trainings, where they achieved near perfect attendance by automatically sending vehicles to CVs homes. In the meetings, the staff used a specific motivational strategy:

Before any meeting of CVs, we told them you are not doing work – *kaj* in Bangla. We told them: “don’t say, ‘I am doing work’ say, ‘I need to practice the modules’” If the word k*aj* comes, the direct linkage with money will happen. So [the mentors and I] replace the word ‘work’ with the word ‘practice.’ (Interview, 6/11/2019).

The PRADAN staff continued that they enthusiastically tell the CVs, “*you are change vector, you are bringing change into your society and you take the responsibility yourself. Nobody told you to take responsibility forcefully.* (Interview, 6/11/2019). The staff explained the whole mentor team used these tactics, in order to emphasize the importance of CV ‘practice’ and forge a sense of personal responsibility and identity.

This strategy to promote voluntarism was effective yet produced an uneven experience of CV work dependent on socio-economic status. CVs reported attending trainings universally and valued them for providing education and social support. Most CVs also had a strong command of the curriculum and felt the community responded to them well. However, this was not universal and the several CVs that struggled to recall the curriculum complained this was because they had much household and agricultural work so they could not devote their full attention to CV work or the trainings. One mentor discussed a struggling CV:

She is busy at her household works. She cannot provide time to this work. Her husband also does not support her….To do this work you need body and mind….so this CV has only one. Whatever is told in the training, she cannot carry it completely in her head….This is the problem. (Mentor interview, 6/2/2019h)

PRADAN staff were cognizant of this situation, stating *“only those CVs who are a little better off from economic points of view are continuing it in a real way”* (PRADAN interview 6/10/2019). This dynamic, where only more socioeconomically privileged women could devote “body and mind” to volunteering, introduced a tension into Purulia’s program since the participatory intervention was designed around eliciting women’s experiential knowledge around poverty, inequality, and nutrition. Using women who may lack such knowledge may have impacted power dynamics and discussions within these modules. (see Nichols, 2021b).

Many CVs who struggled with curriculum recall asserted that if they had payments, they might be feel motivated to remember better. For example, one CV protested, *“as there is no salary, I sometimes forget about the stories. If there would have been salary, then I would have some fear before forgetting.”* (CV interview, 6/8/2019ku). Mentors, too, felt that motivation was a problem and yet they had limited recourse to hold CVs accountable. They conceded that while they strategically called CV work "practice," many CVs did not accept this logic and that if they wanted to solve the problem of motivation the answer was “payment”. (Mentor FGD).

### 5.5 Benefits, costs, equity

Despite concerns around CV motivation, Purulia CVs were able to identify more non-financial benefits from their work than in other sites. Perhaps due to motivational tactics and trainings, several CVs felt that these benefits were sufficient ‘payment’ for their efforts. For example, one CV stated,

Many CVs have raised the payment issue. They [PRADAN] said that through our work, we are improving the health of the people in the village. Then we thought that it is fine. We are getting respect from others. This is also an achievement. (CV interview 6/9/2019a).

Approximately 25% of Purulia CVs shared this woman’s sentiment and felt satisfied doing the work voluntarily because they were helping their community, increasing social status, or gaining a valuable education. The remaining 75% also cited many of these benefits but were unconvinced they alone could be a substitute for honorariums. In one FGD, the women tell us how they have brought the issue of payment several times to PRADAN and always received a similar response,

[Mentors] said that the education is the main part and not the work. I should have told them, “then do your works without salary. Will you be able to do it?” Anyways, I did not tell that. I intended to tell that, but I could not. (CV FGD, 6/8/2019b).

Thus, while Purulia CVs were able to identify more benefits from their work, they also identified more ‘costs’ to their engagement. Women stated trainings were the most difficult component since they had to leave the village for a full day. In an FGD the issue became heated, with one CV saying: *“we have so many works. So going to Purulia for three days only increased our work…with all these works to be done we hoped for an honorarium…but we did not get it so we are a bit unhappy”* (CV FGD, 6/8/2019a). While Bastar CVs simply did not go to trainings to avoid hardship, the greater levels of social capital and supervisorial oversight in Purulia seemed to make ‘skipping’ more unacceptable.

Interestingly, despite their volunteer status, some community members believed CVs to be salaried workers. Approximately one-third of CV respondents said they explained to SHGs they are working voluntarily, but the community accused them of secretly receiving salary (see also Kok et al., 2017). One CV recounted SHG members will say *“you are getting something and so, you are going [for trainings]. If there was no benefit, would you have gone? Without profit, will you do anything?”* (CV FGD 6/14/2019). While mentors stated they clarified women did not receive pay, it seemed this distrust was ongoing. Because many of these women had worked for PRADAN before, there may have been community suspicion of ‘opportunity capture’ where more socio-economically advantaged women hoard benefits of development (e.g., see Mercer, 2002).

Despite these reports of personal sacrifice and mistrust, CVs expressed varying comfort levels raising the payment issue to PRADAN. Many indicated they thought they might receive preferential selection for other remunerated projects, and thus perhaps did not want to damage relations. Approximately 80% of CVs did do other paid work for PRADAN (e.g., group audits), which also may have created reluctance to express discontent. However, most tellingly CVs reported that while many women *individually* complained about payment, they were never able to do so collectively. One CV explained,

When CV have discussion among each other, they tell me to raise this [payment] issue in the meeting. When I raise this issue, then mentors ask “only you are raising this issue. Others are not saying anything.” (CV interview, 6/10/2019)

Thus, while Purulia enacted volunteer tactics that went beyond letting women work when and how they wish – their focus on motivational trainings created a stronger cadre who reported experiencing some psychological empowerment. Yet, while most CVs demonstrated some political empowerment in their ability to analyze and critique their labor arrangements, they were unable to collectively take action to change these relations. Thus, as a final short example, we consider PS.

### 5.6 Paschim Singhbhum – Honorariums and accountability

As a pilot site, PS experimented with different curriculum modalities in 2015 before the program began employing mentors in 2016. Like Bastar, staff faced challenges in recruiting CVs due to the block’s ethno-linguistic diversity and high levels of illiteracy. Moreover, PS is home to ongoing Naxal violence that made mobility and safety a preeminent concern in implementation. Due to these challenges in the pilot phase, they were unable to recruit one CV to cover each hamlet, so they relied on a smaller group of CVs to cover multiple, far-flung hamlets. Like other sites, these women faced resistance from their families to volunteering, yet rather than simply drop out, these women collectively decided to make demands for honorariums. Several CV respondents recounted how it happened:

R2: We talked to each other. Someone asked “We are not getting any money. What should we do? We are also getting scolded in our house.” We also meet at the PRADAN office. We talk to each other before the meeting.

R1: For whole day of work, we were not getting anything. We used to get only allowance for food and travel.

R2: They decided to start paying us. We had told [PRADAN] that “we will not be able to work if we are not given money. Nobody will work for free. If I would have worked as labor, I would have earned something. Here, you ae not giving anything apart from food and travel allowance.” After that, they started paying us. (CV FGD, 6/15/2019)

The CVs success in collectively bargaining may have been the result of being a smaller group (approximately 30, compared to 70 elsewhere) making it easier to create a unified position. They were also operating without mentors, so they arguably had more bargaining power with PRADAN. Finally, as CVs covered SHGs outside of their own hamlets, they had greater justification for payment.

However, even with remuneration, staff faced difficulty keeping CV positions filled. Community-based selection processes continued to yield women with amenable households rather than those that expressed strong motivation. For example, one PS CV explained she did not have interest in the position but was selected because she had no children: *“I told them to select another woman, but nobody wanted to do it, so then I said that I would work.”* (CV FGD, 6/15/2019). While mentors elsewhere believed payment was the answer to CV recruitment and motivation, the PS example revealed that payment may be necessary, but it is not alone sufficient (see also Kok et al., 2015).

### 5.7 Management

Yet, the presence of payments shaped staff attitudes towards CVs in ways not observed elsewhere. While the Purulia block team used assertive motivational tactics to maintain CV accountability and the Bastar team adjusted their expectations, the CVs in PS seemed to face a more stringent system of management because they were paid. For example, one CV reported they were sometimes made aware that they are held to higher expectations and scolded because even though they are paid, there have not been major changes in the community. She recounted:

When we went to training, the trainer told us that we are getting money and other [CVs] are not. Still, results are not good here. They were scolding us. (CV FGD 6/14/2019a)

CVs also reported that PRADAN staff performed “checks” to make sure they are performing work properly.

Sometimes [PRADAN] staff come for checking. They know about the dates of the meetings. They do not call us over phone. They come to the meeting without telling us anything. When we start the meeting, they come afterwards. They check whether we are training other SHG women properly or not. (CV FGD 6/14/2019b)

In Purulia and Bastar, CVs were happy to receive support from PRADAN or mentors, thus it was interesting that what was considered field-level support by CVs in other sites was seen in a more ‘punitive’ fashion once payment was instituted.

While most PS CVs had good command over the curriculum and felt trainings were helpful, they reported their facilitation skills had mostly developed because they had delivered modules to many different groups in an effort to maximize monthly income. While more opportunities for practice improved CV’s facilitation skills, PRADAN staff noted because CVs traveled to outside villages, there were sometimes “communication and acceptance” issues because women came from different communities (PRADAN interview 6/13/2019). Moreover, similar to Purulia, several CVs had poor mastery of stories. PRADAN staff explained several CVs did not work up to par because they were *“paid staff so the interest was little bit less”*. The staff lamented that the payment system was “a problem” and other sites (like Purulia) saw “better results” because they used “volunteers with more interest” (PRADAN interview, 6/13/2019). This was illuminating as it precisely contradicted Bastar and Purulia mentor opinions that CVs’ volunteer status was the principal challenge to motivation, and also because approximately one-third of Purulia CVs who struggled with curriculum claimed that payments might make them ‘remember better’.

### 5.8 Benefits, costs, equity

In discussing the benefits of their work, PS CVs were clear that while the extra income was helpful, they valued other aspects of the work more. More than other sites, PS CVs felt the best incentive was the ability to travel to different villages and meet different women. It was interesting that the professionalization (e.g., moving beyond one’s immediate neighborhood) was what program designers intended to move away from in implementing the ‘CV’ system, yet it was what PS CVs were most drawn to. Consistent with literature on NGO professionalization, the PS change agents did not report increases in social prestige as much as Purulia (Jakimow, 2010). However, similar to Purulia some cited examples of tensions arising due to their payments. One CV noted that women try to leave meetings early:

Then I say ‘do not get out now.’ They [SHG women] say ‘you can do this, because you are getting paid.’ Then I say ‘why are you talking about money? How much we are getting!’ I scold them. then they stay back. (CV FGD 6/14/2019b)

While this data resonates with other findings from CHW programs (Ormel et al. 2019), it is interesting that Purulia CVs also faced resistance despite working voluntarily. This trend illuminates the challenges of payments in resource-poor contexts and highlights the need to ensure paid opportunities are distributed equitably. In PS, we found that even small, timebound income streams could make big impacts on women’s lives. Several PS CVs noted how income from CV work had not just allowed them to improve their household condition, but they were now allowed to move freely in ways they could not before. Thus, while the honorariums were quite meager (about half the daily labor wage rate), one unmarried CV reported she can now "go anywhere" (CV FGD 6/142019a) whereas another said she could now "feed my son good food" (CV FGD 6/15/2019).

Finally, FAAM’s initial motivation to use volunteers was based on a hypothesis that volunteers might continue to work beyond the project end-date since their actions were not tied to payments and they presumably joined due to their personal interest rather than a desire for money or professional advancement. While CVs in all sites agreed they would continue to talk about their learnings in their own SHGs, they all stated they would not go beyond this level of effort. Mentors, however, seemed more dubious and felt that without guidance and supervision, most CVs would not continue discussions. While more research is needed to assess group-based intervention’s sustainability (but see Sondaal et al., 2019), one PS CV aptly illustrated their ultimate dependence on continuous trainings stating *“whatever I know I will tell [to my SHG]. If I do not know something, how will we tell?”* (CV FGD 6/14/2019a).

## 6 Discussion and conclusion

**Fig. 1:**
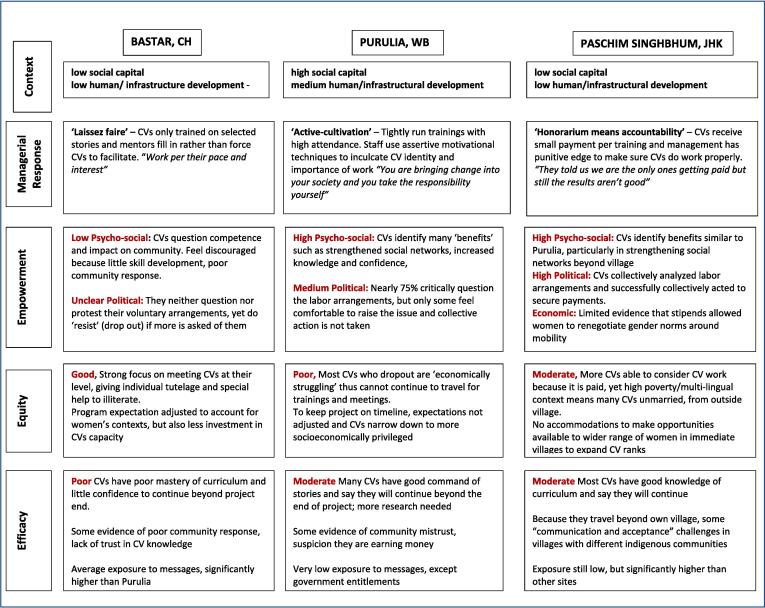
Empowerment, efficacy, and equity effects of differing voluntarism ‘styles’.

This paper sought to answer questions around how context shaped volunteer management and incentivization and how different ‘styles’ of management impacted tradeoffs between volunteer empowerment, equity, and efficacy through developing a tri-partite “typology” of voluntarism models (Fig. 1).

Program-level hypotheses argued that voluntarism was more sustainable because through emphasizing non-material benefits such as knowledge, women would join based on their intrinsic motivations rather than for money. We found that in practice women were nominated due to their household’s willingness to let them volunteer, and while the majority expressed intrinsic motivation for the work, about one-quarter only joined to fulfil their responsibility. This process of selection introduced two central tensions into the program. While voluntarism was meant to attract women with intrinsic motivation, being nominated in a public forum or by an authority figure may have made women feel socially compelled to consent. Second, because women were selected based on their ability to ‘work for free’, selected women were often categorically distinct from their peers in that they had relative socio-economic security or little household responsibility. However, despite selecting women who purportedly had more availability to volunteer, all three sites faced challenges retaining and motivating CVs. In each site, both the teams and the CVs responded to the tensions of voluntarism in unique ways that reflected evolving contexts of social capital and human development.

In terms of context, we build on previous work (Nichols, 2021a) to find both basic socioeconomic development and social capital between the implementing agency (be it government or NGO) and volunteers are critical for effective voluntarism yet acknowledge that relations of reciprocity can also be misused (c.f. Maes, 2012). In Bastar and PS, PRADAN had weaker social ties to the community so requesting timebound active voluntarism was untenable at project inception. In Purulia, PRADAN had been investing in SHG members’ capacities for years, thus has an easier time recruiting women. As many Purulia CVs received other paid work from PRADAN, this may have also increased their propensity to ‘work for free’ in other capacities. Additionally, the ethno-linguistic diversity and histories of Adivasi oppression alongside low literacy rates in Bastar and PS made working across these sites challenging, absent more substantive investments. In Purulia, conversely, the shared language (Bengali) and higher literacy levels were key enablers allowing PRADAN to recruit qualified women.

Previous literature similarly suggests that the presence of a ‘social (or moral) economy’ based on reciprocity and trust versus a ‘market economy’ has a strong impact on whether voluntarism is viable (Glenton et al., 2010, Ormel et al., 2019). We see this to be true in our data, yet our conceptual focus on ethics turns us to Maes et al. (2010), who argues that the presence of a social economy is questionable justification for instituting voluntarism ‘from above’, particularly if the volunteers are women struggling with livelihood security (see also Jakimow, 2010, WHO, 2018). In highlighting the dynamism of rural South Asian ‘context’, Jakimow (2010) questions whether historical notions of *sewa* (community service) are tenable within grassroots NGOs operating in an increasingly monetized rural India. We see these questions come to the fore in our data, as project goals around inculcating a ‘spirit of voluntarism’ oftentimes conflicted with CV desires for secure livelihoods.

Yet our case vignettes do not deliver a tidy answer to the ethics of voluntarism and instead highlight the need to nuance the ‘varieties’ of voluntarism in terms of the ways it balances responsibilities, rewards, and economic, physical, or emotional ‘costs’ of participation (c.f. Baillie-Smith et al., 2020). Bastar arguably, implemented a more ethical form of voluntarism, respecting CV autonomy and adjusting the program to meet CV limitations. This was not their initial approach, but CV resistance to more demanding forms of voluntarism influenced the team to reduce CV responsibilities by ending centralized trainings. While this perhaps made the program more inclusive through offering field-level training, it failed to forge a sense of social identity or enable worker empowerment seen through Kane et al.’s (2016) framework of competence, meaningfulness, and impact. Yet, though the approach did not enable psychological empowerment, women also did not express resentment at their volunteer status.

Conversely, although Purulia’s approach to rigorous centralized trainings and active voluntarism led women to feel competent and see more meaning in their work, it also led to greater inequities within CV ranks as not all women had the ‘body and mind’ to devote to volunteering or attending outside trainings. While many women critiqued their labor arrangements, about 25 % of women were content volunteering, which perhaps hampered more collective action towards securing payment. The staffs’ approach to actively shaping women’s motives is often used in CHW programs but has been critiqued for being counterproductive to empowerment goals of developing a critical consciousness (Closser et al., 2019). Within Purulia this approach was also ethically troubling as many women expressed ambivalence about their volunteer status yet reported feeling unconfident about demanding payments because staff emphasized the many non-financial benefits they had gained. Moreover, while women had a strong command of curriculum, we found this may have not translated into a more efficacious program since they had socio-economic privilege relative to their peers. Such privilege may have hampered more candid discussions around poverty-nutrition linkages or led to community distrust that they were ‘secretly’ being paid.

The PS site was unique because CVs collectively secured honorariums early in the project. The site was illuminating because while Purulia and Bastar mentors felt that ‘payments’ would be the answer to poor CV motivation, PS staff felt their program would be stronger if CVs were volunteers. The payment models, however, meant that staff seemed to use a more stringent style of management that made CVs feel like they were being surveilled rather than supported. Because women were paid per training and had to cover multiple villages, this also led them to work farther afield, despite potential problems with community ‘acceptance’. While we do not have as clear of a picture of equity within PS as the other sites, some CVs reported gaining bargaining power in their household once they started receiving income. This resonates with perhaps the most potent justification for providing remuneration in that it disrupts entrenched gendered norms that frame women’s care work as something that should be provided for free (Steege et al., 2018).

In conclusion, we can draw three main lessons for policy makers from these vignettes. The first is that there is a tension in volunteer programs around selection. While CHW and group-based programs that feature community selection tend to be more successful (Desai et al., 2020, Scott et al., 2018), when the position is unpaid there is risk of putting women into roles, they may feel unable to decline. Moreover, rather than selecting the women who might be able to make the most impact, gain the most from the training, or have strongest intrinsic motivation, women are selected due to logistical reasons such as having ‘a good husband’ or not having children. While voluntarism theoretically attracts women with strong intrinsic motivations (Glenton et al., 2013), we found that in practice it meant women with fewer responsibilities were ‘told’ to volunteer regardless of motivation.

Second, there is a tension in the possibility that greater expectations or responsibilities can lead both to greater intrinsic and extrinsic benefits and to greater ‘costs’ by making women do ‘double duty’ at home or by excluding the more marginalized who cannot devote time to volunteer. Although the laissez faire model did not breed resentment or impinge on women’s time, it also was not very empowering for either CVs or communities. While more responsibility led to greater psychological empowerment gains in Purulia, it also bred resentment among CVs and excluded the economically marginalized. Clearly greater investment in women’s capacities would be required to achieve impacts – particularly in the form of centralized trainings to build networks and engage in participatory learnings – yet many women were unable to give time for such investments without compensation. Here the time issue comes to the forefront in that while the change-agent position was designed to require minimal labor by only working in one’s hamlet, it did not account for the time needed to attend monthly trainings further afield. Thus, future programs might consider offering honorariums or scholarships to attend trainings to open them up to a greater variety of women.

Finally, there is tension in the outsized impact payments have on expectations and management. It is an interesting paradox that while Purulia and Bastar felt payments were the solution to motivation and attrition problems, the PS team felt precisely the opposite. While much CHW literature explains supervisors’ unrest about incentives either distorting intrinsic motivations or conversely making it hard to demand accountability (Singh et al., 2015, Ormel et al., 2019), these case studies question the significance of payments in improving performance. In both PS and Purulia, where trainings were broadly similar, about the same proportion of women struggled with performing their duties. While Purulia CVs argued their performance would improve if they received money, PS staff claimed the reason CVs struggled to remember was because they were more motivated by money. Kane et al. (2016) argue that no matter what the incentive structures, it is disempowering for women when they do not feel appropriately valued. Arguably, in both sites, CVs expressed they were not valued appropriately since PS CVs received less supportive supervision and Purulia CVs resented ‘working for free’. This highlights the need for *both* honoraria and supportive supervision and trainings, where stringent management techniques are avoided and goodwill is fostered through valuing the opportunity cost of women’s time. Thus, perhaps seeing honoraria less a tool to demand accountability, and more as a mechanism to allow for greater inclusivity and to build social capital with communities would be one way to reimagine the role of financial incentives within change-agent type programs.

In sum, while payments may not make an unmotivated volunteer into a motivated one, this analysis suggests payments *would* potentially allow more marginalized women to participate, which may be key to making more equitable and efficacious impacts.

## CRediT authorship contribution statement

**Carly Nichols:** Conceptualization, Methodology, Investigation, Formal analysis, Writing – original draft, Writing – review & editing.

## Declaration of Competing Interest

The authors declare that they have no known competing financial interests or personal relationships that could have appeared to influence the work reported in this paper.

## Data Availability

The data that has been used is confidential.

## Footnotes

1 IFPRI (2018). Data available on request.

2 These ranked CVs by number of modules transacted and subjective appraisals of their ability to engage members and motivate behavior changes.

3 Many, in fact, expressed the wish they could learn more to share it with their groups.
